# Role of Endogenous Cathepsin L in Muscle Protein Degradation in Olive Flounder (*Paralichthys olivaceus*) Surimi Gel

**DOI:** 10.3390/molecules26071901

**Published:** 2021-03-28

**Authors:** Chang Woo Kwon, Pahn-Shick Chang

**Affiliations:** 1 Research Institute of Agriculture and Life Sciences, Seoul National University, Seoul 08826, Korea; rnjsckdd@snu.ac.kr; 2 Department of Agricultural Biotechnology, Seoul National University, Seoul 08826, Korea; 3 Center for Food and Bioconvergence, Seoul National University, Seoul 08826, Korea; 4 Center for Agricultural Microorganism and Enzyme, Seoul National University, Seoul 08826, Korea

**Keywords:** olive flounder (*Paralichthys olivaceus*), surimi, cathepsin L, protein degradation, cathepsin L inhibition

## Abstract

We investigated the effect of endogenous cathepsin L on surimi gel produced from olive flounder (*Paralichthys olivaceus*). The amino acid sequences of six proteins predicted or identified as cathepsin L were obtained from the olive flounder genome database, and a phylogenetic analysis was conducted. Next, cathepsin L activity toward *N*-α-benzyloxycarbonyl-l-phenylalanyl-l-arginine-(7-amino-4-methylcoumarin) (Z-F-R-AMC) was detected in crude olive flounder extract and a crude enzyme preparation. A considerable decrease in the level of myosin heavy chain (MHC) in surimi occurred during autolysis at 60 °C. In contrast, the levels of actin, troponin-T, and tropomyosin decreased only slightly. To prevent protein degradation by cathepsin L, a protease inhibitor was added to surimi. In the presence of 1.0% protease inhibitor, the autolysis of olive flounder surimi at 60 °C was inhibited by 12.2%; the degree of inhibition increased to 44.2% as the inhibitor concentration increased to 3.0%. In addition, the deformation and hardness of modori gel increased as the inhibitor concentration increased to 2.0%. Therefore, cathepsin L plays an important role in protein degradation in surimi, and the quality of surimi gel could be enhanced by inhibiting its activity.

## 1. Introduction

Olive flounder (*Paralichthys olivaceus*), also known as the bastard halibut, is widely consumed raw in East Asia because of its high levels of nutrients and mild taste. Artificial cultivation has been introduced to satisfy the high demand for olive flounder in the Republic of Korea and Japan. After the development of seed production technology by the Fishery Research Institute at Kinki University in 1965, cultivation of olive flounder was transferred to public farmers from 1970 onwards [1]. Finally, a practical cultivation method was developed in 1980 based on artificial seed production technology [2]. However, changes in consumption trends and trade conflicts have occasionally led to oversupply of olive flounder to local fish markets. Moreover, despite falling demand for olive flounder, farming productivity has increased because of improvements in cultivation technology. To overcome these problems and provide economic support to farmers, a number of studies on processed olive flounder products, such as steak, terrine, cutlets, surimi gel, and noodles, have been conducted [3,4,5,6,7]. To date, most studies have focused on the quality and commercial value of surimi gel.

Surimi gel is a three-dimensional protein structure typically produced from deboned fish flesh. Formation of surimi gel is termed setting (suwari in Japanese), which refers to the polymerization of myosin heavy chain (MHC) by endogenous transglutaminase [8,9,10]. However, when surimi is exposed to temperatures of 40–60 °C, its protein components, particularly MHC, may be degraded. The resulting weak gel tends to disintegrate, reducing product quality and value; this is known as the modori phenomenon [11,12]. The degradation is caused by endogenous proteases in fish muscle, particularly cysteine- and serine-type proteases [13,14,15,16]. As well as cathepsin B and H, cathepsin L is the major protease responsible for modori—it still remains in surimi after intensive washing and bleaching—and exhibits high proteolytic activity in several fish species [17,18,19]. Moreover, degradation of olive flounder proteins occurs postmortem during refrigerated storage [20].

To enhance the strength of surimi gel by inhibiting endogenous proteases, a number of food additives are used [21,22,23]. However, the use of blood plasma renders the surimi product off-color and severely impairs the flavor. To solve this problem, microbially expressed protein additives (such as chicken and pineapple cystatin) are used to prevent the autolysis of mackerel surimi and Japanese tope shark (*Hemitriakis japanica*) surimi [24,25].

The olive flounder is an economic marine resource to produce kamaboko. However, little information is known about the role of endogenous cathepsin L in the autolysis of olive flounder, resulting in poor gel quality and reduced commercial value. To overcome these problems and produce marketable products, the development of practical preservation and processing technology for surimi is necessary. Therefore, in this study, we examined the autolytic patterns, activity of cathepsin L, and its inhibition with a protein inhibitor during production of surimi gel.

## 2. Results and Discussion

### 2.1. Identification of Cathepsin L and Its Inhibition Mechanism

**Figure 1:**
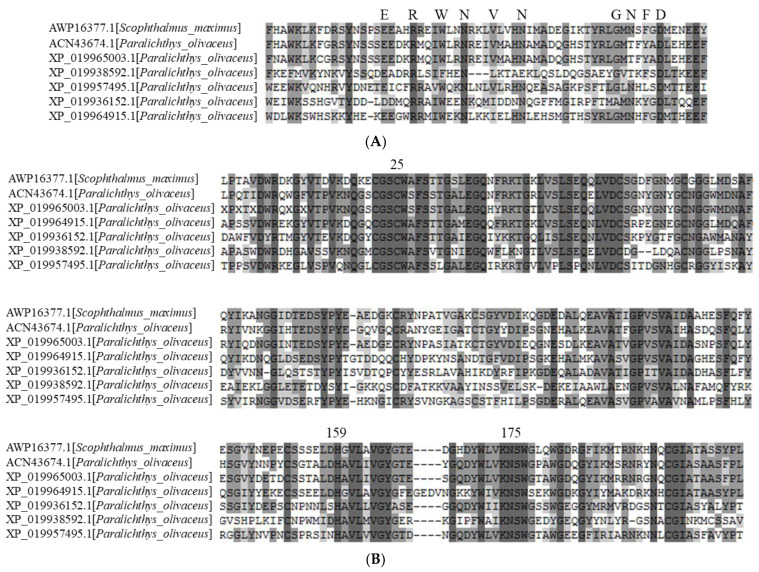
Multiple alignments of the deduced amino acid sequences of cathepsin Ls (olive flounder) with turbot. The deduced amino acid sequences of the inhibitor domain (**A**) and peptidase domain (**B**) were aligned by ClustalW. Identical and conserved amino acid residues are darkly shaded. Conserved signatures (ERWNVN and GNFD) and catalytic triad residues (C, H, and N) are highlighted in bold and indicated above the alignment.

The amino acid sequence deduced from the mRNA sequence of liver-specific cathepsin L1 was used to carry out a BLASTP search. Five proteins predicted as cathepsin L were obtained from the olive flounder genome database. These proteins contained the highly conserved ERF(W)NI(V)N (EXXXRXXXFXXNXXXIXXXN) motif in the long alpha-helix structure of the propeptide, and a GNFD (GXNXFXD) motif at the kink of the beta-sheet prior to the short alpha-helix (Figure 1A). The slight variation in the ERFNIN-GNFD motif suggests a relationship with the I29 inhibitor of the cathepsin L group and is distinct from the cathepsin B subfamily. As in all cathepsin L–like proteins, there were conserved residues associated with the catalytic triad—namely, Cys25, His159, and Asn175 (papain numbering)—as well as six cysteine residues involved in the formation of the three disulfide bonds (Figure 1B). Cys25 is located within a highly conserved peptide sequence (CGSCWAFS). His159 is proximal to amino acid residues of low molecular weight, such as glycine and alanine. Asn175 is part of the Asn-Ser-Trp motif. The cathepsin L propeptide inhibitors possess a compact domain N-terminal region comprised of two α-helices that cross each other, stabilized by the three Trp or aromatic residues. Studies have shown the importance of these aromatics in maintaining the function and structural integrity of the propeptide [26]. There is also a stretched and flexible region of the propeptide that functions to cover the groove between the two domains of the mature enzyme, while a third shorter α-helix sits in the active site cleft. The C-terminus of the propeptide sits on the surface, parallel to the mature enzyme, forming hydrogen bonds with the S subsite; the N-terminal residues form hydrogen bonds with the S’ subsite. The propeptide thus blocks substrate access to the active site, and the reverse orientation helps save the propeptide from proteolysis.

### 2.2. Cathepsin L Activity

Cathepsin L is a major protease involved in the degradation of myofibrillar proteins resulting from the pH decrease in postmortem stages. To confirm the presence of cathepsin L in olive flounder, a crude enzyme solution was obtained from the crude extract and the specific activity was determined from the hydrolysis of *N*-α-benzyloxycarbonyl-l-phenylalanyl-l-arginine-(7-amino-4-methylcoumarin) (Z-F-R-AMC), which is known for cathepsin L substrate (Table 1). However, Z-F-R-AMC is likely to be cleaved by other cathepsins, such as B, K, and S [27,28,29]. Therefore, the crude extract and crude enzyme were exposed to acidic conditions to deactivate cathepsin B [30]. The activities of cathepsins K and S in fish muscle are considered negligible because the substrate is less sensitive to these proteases than to cathepsin L. The cathepsin L assay indicated that cathepsin L exists in the muscle and could weaken the gel strength of the surimi. Previous studies of the gene sequence and substrate specificity also support the presence of cathepsin L in olive flounder extract [31,32]. The relative activity among cathepsins based on its substrate specificity indicated that the activity of cathepsin L was lower than that of the other cathepsins. However, compared with other studies on cathepsin L, the results may have been affected by the extract preparation, assay conditions, or proenzyme activation steps because procathepsins possess propetides that can be cleaved by autocatalytic processing under specific conditions and cathepsins have a preferred cleavage site of substrates, but they hydrolyze similar amino acid sequences.

### 2.3. Optimum Temperature for the Autolysis of Olive Flounder Surimi

**Figure 2:**
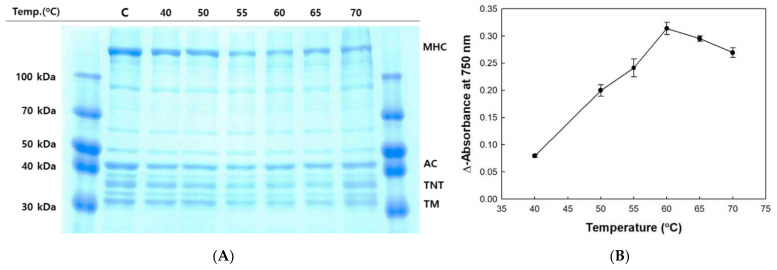
Autolytic pattern (**A**) and trichloroacetic acid (TCA)-soluble peptide content (**B**) of olive flounder paste with increasing temperature. Myosin heavy chain (MHC); actin (AC); troponin-T (TNT); tropomyosin (TM); control (C).

The disintegrated protein profile of olive flounder surimi was measured during incubation at 40–70 °C for 60 min (Figure 2A). The band intensity of MHC, troponin-T, and tropomyosin was reduced significantly as the temperature increased to 60 °C, but a slight reduction in band intensity of actin was observed. The degradation of MHC in surimi was greatest at 60 °C, as indicated by increased soluble protein concentration and the lowest band intensity (Figure 2B). A considerable decrease in the MHC level was observed after incubating surimi at 55–60 °C. However, the degraded soluble protein concentration was lower at 65 and 70 °C, likely due to the denaturation of proteases. Disulfide bonds often cause heat-induced irreversible denaturation because free thiol groups generated by the destruction of disulfide bonds under high temperature conditions cause disulfide bond scrambling. Therefore, temperature is a crucial determinant of autolysis of olive flounder and is correlated with the optimal temperature ranging from 50 to 60 °C for the activity of cathepsin L from other fish species such as arrowtooth flounder (*Atheresthes stomias*) and carp (*Cyprinus carpio*) [12,33].

### 2.4. Autolysis Profile of Olive Flounder Surimi

**Figure 3:**
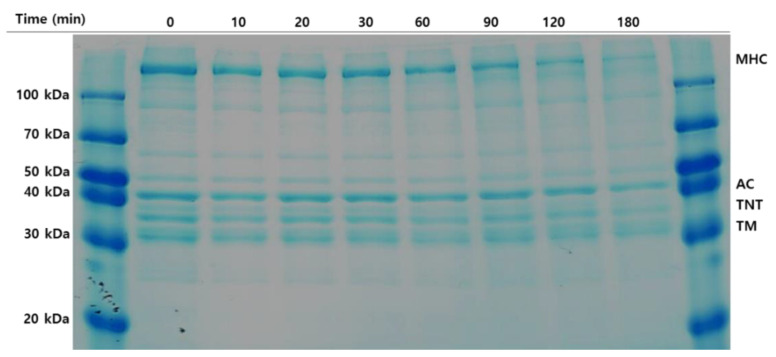
Autolytic pattern of olive flounder paste. Samples were incubated for 180 min at 60 °C. Myosin heavy chain (MHC); actin (AC); troponin-T (TNT); tropomyosin (TM).

Protein degradation of olive flounder surimi was induced at 60 °C and monitored for up to 180 min (Figure 3). MHC and actin were the major myofibrillar proteins in surimi, followed by troponin and tropomyosin. The reduced band intensity of MHC among the myofibrillar proteins was clearly observed with increasing incubation time. A considerable decrease in MHC was observed after incubation for 120 min and the MHC band disappeared at 180 min. The time course of protein degradation differs from the reported complete hydrolysis of MHC when Pacific whiting surimi is heated at 60 °C for 30 min. The disparity may be caused by differences in muscle strength and the content of cathepsin L between olive flounder surimi and Pacific whiting surimi because cathepsin B, cathepsin H, and other proteases are able to be removed by the water washing process. Degradation of troponin-T and tropomyosin increased with time and continued until the end of incubation, but much less degradation was observed macroscopically than that of MHC. A slight reduction in band intensity of actin was evident during incubation for 180 min, implying it is more resistant to protein degradation induced by proteolytic enzymes than other myofibrillar proteins. Those myofibrillar proteins are likely hydrolyzed to smaller peptides or free amino acids showing hydrophobicity with sufficient time, resulting in taste changes in the final surimi products. Therefore, autolysis inhibition of surimi is necessary to improve taste as well as gel strength.

### 2.5. Inhibition of Autolysis Caused by Cathepsin L

**Figure 4:**
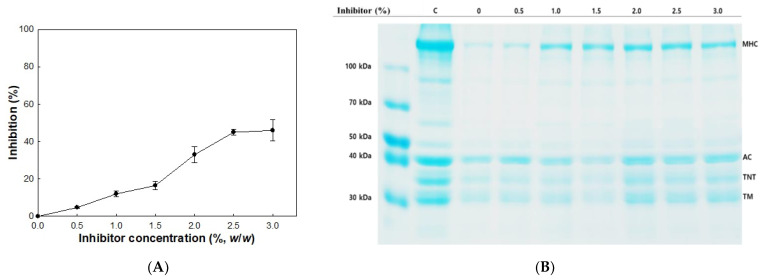
Inhibitory activity (**A**) and autolytic pattern (**B**) of olive flounder surimi with cathepsin L inhibitor. Samples were incubated for 120 min at 60 °C. Myosin heavy chain (MHC); actin (AC); troponin-T (TNT); tropomyosin (TM); control (C).

A cathepsin L inhibitor showed inhibitory activity in the autolysis of olive flounder surimi to different degrees. Generally, the rate of autolysis inhibition was dependent on the amount of inhibitor used, and increased inhibition was observed with increasing concentration of inhibitor (Figure 4A). Autolysis of olive flounder surimi at 60 °C was inhibited by 12.2% in the presence of 1.0% cathepsin L inhibitor. The inhibition of autolysis was maximized up to 44.2% as the cathepsin L inhibitor concentration increased up to 3.0%.

Autolytic patterns of olive flounder surimi incubated at 60 °C for 120 min in the presence and absence of cathepsin L inhibitor at different concentrations are shown in Figure 4B. The reduced band intensity of MHC was partially recovered with increasing concentration of the cathepsin L inhibitor, while the band intensities of actin, troponin-T, and tropomyosin were fully recovered. These results indicate cathepsin L of olive flounder plays an important role in the degradation of MHC and the addition of cathepsin L inhibitor could be an effective way to prevent the degradation of myofibrillar proteins from autolysis.

Protein inhibitors are bio-friendly and low-toxicity materials that can be used in the food industry. However, their higher molecular weight compared to other chemical inhibitors means that their interaction with endogenous enzymes is hampered by connective tissue. Indeed, inhibitory activity was lowered, compared to E-64, when using whey protein concentrate, cystatin, and chicken plasma protein [34,35,36,37]. Therefore, inhibitory activity could be affected by sample preparation, e.g., cutting, chopping, mincing, and mixing. The activity of cathepsin L remaining after the washing treatment for mincing indicates how deeply it is located from the surface of the muscle tissue, unlike cathepsins B and H. In addition, the protein inhibitor could be a substrate for other proteases in fish muscle, as it has many cleavage sites, resulting in the prevention of hydrolysis in myofibrillar proteins.

### 2.6. Effect of Cathepsin L Inhibitor on Textural Properties

**Figure 5:**
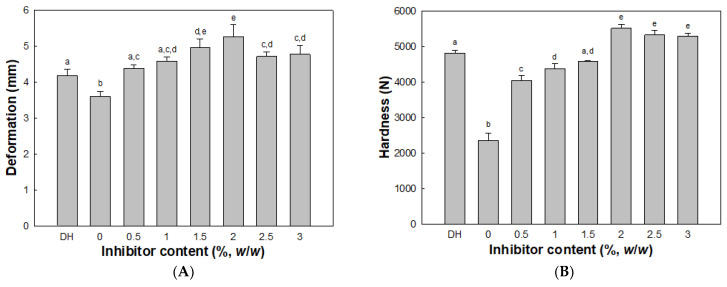
Deformation (**A**) and hardness (**B**) of surimi gels with inhibitor. Different letters indicate significant differences (*p* < 0.05). Directly heated (DH) gel without inhibitor.

The deformation and hardness of modori gels from olive flounder surimi increased as the concentration of cathepsin L inhibitor increased (Figure 5). The deformation and hardness peaked at 2.0% cathepsin L inhibitor, whereas the modori gel showed the lowest deformation and hardness in the absence of cathepsin L inhibitor. At 3.0% cathepsin L inhibitor, the deformation and hardness of the modori gel increased by 76% and 134%, respectively, compared to the control modori gel (without cathepsin L inhibitor). Both deformation and hardness of modori gels decreased at 2.5% and 3.0% cathepsin L inhibitor when compared to those of 2.0% cathepsin L inhibitor. These results were similar to those of a previous study in Pacific whiting and walleye pollock and could be explained by the dilution effect of MHC constructing a strong gel network in surimi [35,36]. On the other hand, a directly heated gel exhibited greater deformation and hardness compared to the control modori gel because of different incubation conditions. It was evident that cathepsin L weakening the surimi gel network should be controlled with inhibitors during storage or the surimi must be processed immediately.

### 2.7. Effect of Cathepsin L Inhibitor on Surimi Gel Whiteness

Whiteness is one of the most important quality indicators for surimi products because surimi gels with high whiteness are preferred by consumers [38]. No difference in whiteness (25.72 ± 0.6) was observed between samples with and without cathepsin L inhibitor. Therefore, cathepsin L inhibitor could be added without adversely affecting surimi gel whiteness.

## 3. Materials and Methods

### 3.1. Materials

Dimethyl sulfoxide (DMSO), Trizma^®^ base (≥99.9%), *N*,*N*,*N*′,*N*′-tetramethylethylenediamine, acetic acid (≥99%), 2-mercaptoethanol (≥99%), sodium dodecyl sulfate (SDS), glycerol (≥99%), glycine (≥99%), Z-F-R-AMC, 7-amino-4-methylcoumarin (AMC), and TCA were purchased from Sigma-Aldrich (St Louis, MO, USA). Coomassie Brilliant Blue R-250 was purchased from Bio-Rad (Hercules, CA, USA). Olive flounder was purchased from Noryangjin Fisheries Wholesale Market in Seoul, Korea. Cathepsin L protein inhibitor was obtained according to the method described by Kwon et al. [39].

### 3.2. Identification and Phylogenetic Analysis of Cathepsin L

To obtain information about cathepsin L in olive flounder, the amino acid sequence of cathepsin L1 reported by Kim et al. [31] was used to query the NCBI non-redundant protein sequence database (http://www.ncbi.nlm.nih.gov, accessed on 2 February 2021). The amino acid sequences from olive flounder and other species were aligned using ClustalW software to confirm the conserved domains and construct a phylogenetic tree of cathepsin L. Next, a phylogenetic analysis was conducted using the maximum-likelihood method in MEGA software (version 10.0; https://www.megasoftware.net, accessed on 2 February 2021). Bootstrapping with 1000 replications was performed to evaluate the phylogenetic tree.

### 3.3. Surimi Preparation

Olive flounder was eviscerated, beheaded, deboned, and skinned. Next, each fillet was cut into thin slices (thickness ~ 1 cm) and minced in a blender at 4 °C. The fillet paste was passed through a strainer with a mesh size of 1 mm at 4 °C to eliminate residual pin bones and scales.

### 3.4. Preparation of Crude Enzyme

The strained fillet paste (surimi) was suspended in 25 mM sodium phosphate buffer (pH 6.0) at 4 °C. After centrifuging the mixture solution at 14,000× *g* for 30 min, the supernatant (crude extract) was filtered through a 3-μm membrane filter. Next, crude protein was precipitated in ammonium sulfate solution at 100% saturation. After centrifuging the mixture solution at 14,000× *g* for 30 min at 4 °C, the precipitate was dissolved in 25 mM sodium phosphate buffer (pH 6.0) and dialyzed against the same buffer to remove remnant ammonium sulfate.

### 3.5. Cathepsin L assay

A crude extract and crude enzyme preparation was exposed to 25 mM formic acid at pH 3.0 and incubated for 5 min at 25 °C to activate the proenzyme of cathepsin L and deactivate cathepsin B [30]. Next, the activated crude samples were mixed with sodium acetate–acetic acid buffer, resulting in a final pH of 5.0. The enzyme reaction was initiated by adding Z-F-R-AMC dissolved in dimethyl sulfoxide solution. Activity was determined by measuring the liberated AMC (excitation wavelength of 355 nm, emission wavelength of 460 nm) from the Z-F-R-AMC (fluorogenic substrate) using an automated microplate spectrofluorometer (SpectraMax M2e; Molecular Devices, Sunnyvale, CA, USA). One unit of enzyme activity was defined as 1 nmol AMC released per minute at 30 °C.

### 3.6. Autolytic Activity Assay

An autolysis assay was conducted according to the method described by Morrissey et al. [40]. Surimi (3 g) with and without cathepsin L inhibitor was put in a glass beaker and heated in a water bath at various temperatures and times. Next, 27 mL of 5% (*w*/*v*) TCA stored at 4 °C was added to the beaker to stop the autolysis reaction. The mixture was blended thoroughly and homogenized using an Ultra Turrax T25 (Ika Werke GmbH & Co., Staufen, Germany) at 11,000 rpm for 1 min and then stored in a refrigerator. Then, the homogenate was centrifuged at 6000× *g* for 10 min. The amount of TCA-soluble peptides in the supernatant was measured according to the method described by Lowry et al. [41]. The degree of autolysis inhibition was determined as follow Equation (1):

% Inhibition = (A − B) × 100 × A(1)

where A is the TCA-soluble peptide content in a sample without cathepsin L inhibitor, and B is the TCA-soluble peptide content in a sample with cathepsin L inhibitor.

### 3.7. Sodium Dodecyl Sulfate–Polyacrylamide Gel Electrophoresis

Protein degradation of surimi was determined by monitoring protein band intensity on gel electrophoresis. To solubilize the integrated or the disintegrated proteins, 27 mL of 5% SDS (85 °C) was added to the incubated surimi samples. Then, the mixtures were mixed at 11,000 rpm for 1 min using an Ultra Turrax T25, followed by incubation at 85 °C for 60 min. The homogenate was centrifuged at 5000× *g* for 5 min, and the supernatant was subjected to SDS-polyacrylamide gel electrophoresis (SDS-PAGE) according to the method of Laemmli [42]. Supernatants were mixed at a 1:1 (*v*/*v*) ratio with sample buffer (0.5 M Tris–HCl [pH 6.8] containing 20% glycerol, 4% SDS, and 10% 2-mercaptoethanol) and placed in boiling water for 3 min. The samples (20 µg of protein) were loaded onto the polyacrylamide gel (4% stacking gel and 10% running gel) and run at a constant current of 20 mA per gel. Next, the proteins separated on the polyacrylamide gel were stained with a staining solution of Coomassie Brilliant Blue R-250, 7.5% (*v*/*v*) acetic acid, 50% (*v*/*v*) methanol, and 42.5% (*v*/*v*) water and then destained with a destaining solution of 7.5% (*v*/*v*) acetic acid, 50% (*v*/*v*) methanol, and 42.5% (*v*/*v*) water.

### 3.8. Preparation of Surimi Gel

Surimi with a moisture content of 78% (*w*/*w*) was combined with lyophilized cathepsin L inhibitor powder at 0, 0.1, 0.3, 0.5, 1.0, 2.0, and 3.0% *w*/*w* and with sodium chloride (2.5%, *w*/*w*) before being blended thoroughly. The surimi was stuffed into a low-density polyethylene casing (2.5 cm in diameter), and the molded surimi was sealed tightly with polyvinyl chloride wrapping film. The surimi was incubated at 60 °C for 2 h, followed by heating at 90 °C for 20 min in a water bath. After incubation, the gel samples were immediately chilled in ice for 30 min and stored in a refrigerator (4 °C) for 12 h before a texture test. The gel was referred to as “modori gel.” A directly heated gel (DH) was prepared by incubating surimi at 90 °C for 20 min in a water bath.

### 3.9. Texture Profiling

A texture profile analysis of modori gel was conducted using a TA-XT2 Texture Analyzer (Stable Micro Systems, Godalming, UK) equipped with a cylindrical probe (50 mm in diameter). The modori gel was sliced into a cylindrical form (2.5 cm length) to flatten both contact surfaces of the gel and kept at room temperature (25–30 °C). Hardness (N) and springiness (mm) of cylindrical samples were measured using a texture analyzer at the 60% compression and a depression speed of 60 mm/min.

### 3.10. Determination of Whiteness

Color measurement of four gel samples per treatment was carried out using the HunterLab ColorFlex colorimeter (Hunter Associates Laboratory, Reston, VA, USA). The instrument was calibrated each time with a white reference tile and a black reference tile prior to measurements. The color values were expressed as CIE L*, a*, and b*, and whiteness was calculated using the following Equation (2):

Whiteness = 100 − [(100 − L*)^2^ + (a*)^2^ + (b*)^2^ ]^1/2^ (2)

where L* is whiteness or brightness/darkness, a* is redness/greenness, and b* is yellowness/blueness.

### 3.11. Statistical Analysis

All experiments were repeated three times. Data were subjected to analysis of variance and Duncan’s multiple range test using SPSS software (version 23; SPSS Inc., Chicago, IL, USA). *p*-values < 0.05 were taken to indicate statistical significance.

## 4. Conclusions

Postmortem autolysis in olive flounder surimi is highly correlated with the activity of endogenous cathepsin L and the storage temperature. To prevent protein degradation, which reduces gel quality and its commercial value, a cathepsin L inhibitor was added to surimi. The deformation and hardness of modori gels recovered as the cathepsin L inhibitor concentration increased to 2.0%. Therefore, we suggest that inhibition of cathepsin L is essential and practical for producing high-quality surimi gel.

## Figures and Tables

**Table 1:** Cathepsin L activity of crude extract and crude enzyme from olive flounder.

Step	Total Protein (mg)	Total Activity (units)	Specific Activity (units/mg)	Yield (%)	Purification Fold
Crude extract	1682	2103	0.8	100	1.00
Crude enzyme	1533	1686	1.1	91	1.32

## Data Availability

The data presented in this study are available in the article.

## Sample Availability

Cathepsin L protein inhibitor samples are available from the authors.
